# New Cytotoxic Sesquiterpenoids from *Siegesbeckia glabrescens*

**DOI:** 10.3390/molecules20022850

**Published:** 2015-02-10

**Authors:** Qian Wu, Hua Li, So Yoon Lee, Hwa Jin Lee, Jae-Ha Ryu

**Affiliations:** 1 Center for Cell Fate Control and College of Pharmacy, Sookmyung Women’s University, Seoul 140-742, Korea; E-Mails: wuqiansy@hotmail.com (Q.W.); cooldog227@hotmail.com (H.L.); miyuu_chan@naver.com (S.Y.L.); 2 Department of Natural Medicine Resources, Semyung University, Chungbuk 390-711, Korea

**Keywords:** *Siegesbeckia glabrescens*, compositae, sesquiterpenoids, cytotoxicity

## Abstract

Two new sesquiterpenoids, siegenolides A (**1**) and B (**2**), and two known sesquiterpenes **3** and **4** were isolated from *Siegesbeckia glabrescens*. Their structures were elucidated by spectroscopic analyses, and they were further evaluated for their cytotoxic activities against human cancer cells (MCF-7, AsPC-1, SW480, HCT 116, HepG2, HeLa). Compounds **1**–**4** showed differential cytotoxic effects on the target cancer cells with IC_50_ values in the range of 0.9–33.3 μM.

## 1. Introduction

*Siegesbeckia glabrescens* Makino (Compositae) is an annual herb that has been used as a Chinese medicine to treat inflammatory disease, asthma, paralysis and allergic disorders [1]. Flavonoids [1], sesquiterpene [2] and kaurane diterpenes [3] have been isolated from this plant in previous phytochemical studies. It has been reported that the extracts of *S. glabrescens* exhibit antioxidative, anti-inflammatory [1], antiallergic [4], antibacterial [5] and anti-tumor activities [6,7]. In our screening program to discover new antitumor agents from medicinal herbs, two new compounds **1**–**2** and two known sesquiterpenes **3**–**4** were isolated from the ethyl acetate (EtOAc)-soluble fraction of the methanolic extract of *S. glabrescens* by chromatographic procedures. In this study, we present the structural elucidation of these two new compounds, as well as their cytotoxic activities against several human cancer cell lines including MCF-7, AsPC-1, SW480, HCT 116, HepG2 and HeLa.

## 2. Results and Discussion

**Figure 1:**
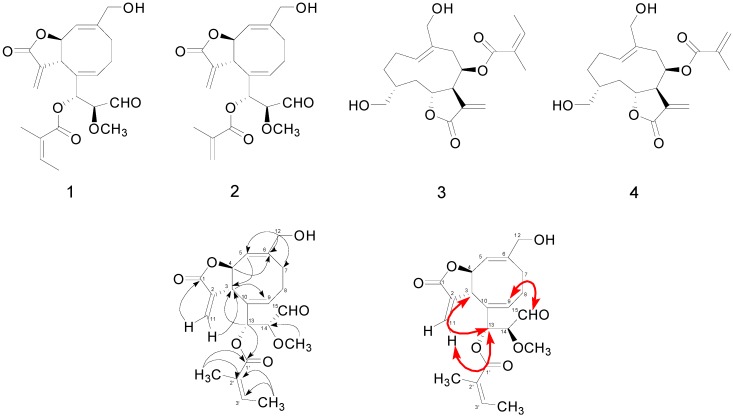
Chemical structures of compounds **1**–**4** and key HMBC (→) and NOESY (bold ↔) correlations for compound **1**.

Dried *S. glabrescens* was extracted with methanol. The crude methanol extract was subjected to liquid-liquid partition as well as a combination of several column chromatography steps to yield two new sesquiterpenoids **1** and **2** together with two known sesquiterepnoids **3** and **4** (Figure 1).

Compound **1** was obtained as an amorphous solid. The HREIMS spectrum suggested a molecular formula of **1** as C_21_H_26_O_7_. The ^1^^3^C-NMR, DEPT, and HSQC spectra showed twenty-one carbon signals including three carbonyl carbons, eight olefinic carbons, three methylene carbons, four methine carbons, one methoxyl and two methyl groups. In ^1^H-^1^H COSY spectrum, H-4 (δ 5.26) correlated with H-3 (δ 2.82) and H-5 (δ 5.09), and also H_2_-8 (δ 2.62~2.73) correlated with methylene protons of H_2_-7 (δ 2.06 and 2.78) and H-9 (δ 6.97). This data suggested that this compound has AMX and A_2_M_2_X spin systems. In HMBC spectrum, oxy-methylene protons H_2_-12 (δ 4.36 and δ 4.39) correlated with C-5 (δ 129.4), C-6 (δ 142.3) and C-7 (δ 33.3), and H-3 (δ 2.82) correlated with C-9 (δ 159.7) and C-10 (δ 141.5) (Figure 1 and Table 1). These data indicated that compound **1** has eight-membered ring in the structure with two double bonds. The correlation between terminal methylene H_2_-11 (δ 5.75 and 6.15) and a carbonyl carbon C-1 (δ 171.2) and C-3 (δ 51.9) in HMBC indicated that compound **1** is a bicycle [6.3.0]-γ-lactone having an exocyclic double bond in lactone ring. We also found the HMBC correlations between oxy-methine H-4 and C-6, and between H-5 and C-3. In ^1^H-^1^H COSY spectrum, we found another AMX spin system from the correlations of H-14 (δ 3.94) with H-13 (δ 6.63) and H-15 (δ 9.43). From the coupling constant of H-15 (*J* = 2.0 Hz) and chemical shift of C-15 (δ 196.8) and the HMBC correlations between H-15 (δ 9.43) and C-14 (δ 79.4), we identified the presence of an aldehyde group that is linked to C-14. In HMBC spectrum, we also found correlation between a methoxyl protons (δ 3.10) and C-14 (δ 79.4), and correlation between H-13 (δ 6.63) and C-3 (δ 51.9). The long range allylic coupling was observed between H-13 (dd, 8.4, 1.2 Hz) and H-9. These results indicated that an oxy-carbon C-13 is linked to the eight-membered ring at C-10. From the coupling constant between H-3 and H-4 (*J = *10.0 Hz,) we postulated the configurations of H-3 and H-4 based on Karplus relationship and the reported values of several bicycle [6.3.0]-γ-lactone derivatives (Figure 1 and Table 1) [8]. Furthermore, the presence of 2-methylbut-2-enoyl group was recognized by ^1^H-^1^H COSY correlation of an olefinic methine H-3' (δ 6.10) with methyl protons (δ 1.93) and the HMBC correlations of methyl protons (δ 1.88) with C-2' (δ 128.9) and C-1' (δ 168.4), and the correlations of methyl protons (δ 1.93) with C-2' (δ 128.9) and C-3' (δ 138.9). The HMBC correlation of an oxy-proton H-13 with carbonyl ester C-1' confirmed the esterification of 2-methylbut-2-enoyl group at C-13. We found NOESY correlations of H-13 (δ 6.63) with H-3 (δ 2.82) and H-11a (δ 6.15) that indicates the orientation of H-13 as β that is located close to H-3 and H-11a. NOESY correlations was also observed between H-9 (δ 6.97) and H-15 (δ 9.43) implying the orientation of methoxyl group as β. This orientation was confirmed by the coupling constant between H-13 and H-14 as 8.2 Hz. Thus, compound **1** was identified as 2-methylbut-2-enoic acid 1-(8-hydroxymethyl-3-methylene-2-oxo-2,3,3a,6,9a-hexahydro-cycloocta[b]furan-4-yl)-2-methoxy-3-oxo-propyl ester. This structure is new and we named compound **1** as siegenolide A.

Compound **2** was obtained as an amorphous solid. The HREIMS spectrum suggested a molecular formula as C_20_H_24_O_7_. The ^1^^3^C-NMR, DEPT, and HSQC spectra showed similar signals as those of compound **1** except showing one terminal olefinic methylene signals of H_2_-3' (δ 5.65, 6.14) instead of the methyl protons (H_3_-4') of compound **1**. The relative stereochemistry of compound **2** was determined by the analysis of NOESY spectra and coupling constants, which was same as compound **1**. Thus, the structure of compound **2** was determined as 2-methyl-acrylic acid 1-(8-hydroxymethyl-3-methylene-2-oxo-2,3,3a,6,9a-hexahydro-cycloocta[b]furan-4-yl)-2-methoxy-3-oxo-propyl ester, which was a new structure and named as siegenolide B.

Compounds **3** and **4** were identified as 2-methylbut-2-enoic acid,2,3,3a,4,5,8,9,10,11,11a-decahydro-6,10-bis(hydroxymethyl)-3-methylene-2-oxocyclodeca[b]furan-4-yl ester (**3**) and 2-methylacrylic acid, 2,3,3a,4,5,8,9,10,11,11a-decahydro-6,10-bis(hydroxymethyl)-3-methylene-2-oxocyclodeca[b]-furan-4-yl ester (**4**), respectively, by comparison with the reported spectral data (Figure 1) [2,9].

The four sesquiterpenoids **1**–**4** were evaluated for their cytotoxic activity on human cancer cell lines such as MCF-7, AsPC-1, SW480, HCT 116, HepG2 and HeLa cells. Compounds **1**–**4** showed differential cytotoxic effects on these cancer cell lines (Table 2). All of them showed significant cytotoxicity against SW480 cell line, with IC_50_ values of 1.8, 0.9, 5.2 and 3.8 μM, respectively. The cytotoxicity of compounds **3** and **4** against AsPC-1 cells was more potent (IC_50_ values of 7.3 and 4.9 μM, respectively) than that of compounds **1** and **2** (IC_50_ values 14.5 and 12.1 μM, respectively). 

**Table 1:** NMR Spectroscopic data (400 MHz, CD_3_OD) for siegenolides A (**1**) and B (**2**).

Position	Siegenolide A (1)			Siegenolide B (2)		
	δ_C_	δ_H_ (*J* in Hz)	HBMC *^a^*	δ_C_	δ_H_ (*J* in Hz)	HBMC *^a^*
1	171.2			171.3		
	136.8			136.7		
3	51.9	2.82, m	9, 10	52.0	2.83, m	9
4	75.7	5.26, t (10.0)	6	75.7	5.33, t (10.0)	
5	129.4	5.09, d (10.0)	3	129.5	5.09, d (10.0)	7, 12
6	142.3			142.2		
7a	33.3	2.78, m		33.5	2.78, m	
7b		2.06, td (12.4, 2.0)			2.06, td (12.4, 2.0)	
8	28.4	2.62~2.73, m		28.5	2.74~2.68, m	
9	159.7	6.97, dd (10.4, 7.6)		159.6	6.97, dd (10.4, 7.6)	
10	141.5			141.6		
11a	121.5	6.15, d (3.2)	1, 3	121.4	6.13, d (3.2)	1, 3
11b		5.75, d (3.2)	1, 3		5.73, d (3.2)	1
12a	60.8	4.39, brd (13.2)	5, 6, 7	61.0	4.39, brd (13.2)	5, 6, 7
12b		4.36, brd (13.2)	5, 6, 7		4.33, brd (13.2)	5, 6, 7
13	70.6	6.63, dd (8.4, 1.2)	3, 1'	71.6	6.56, dd (8.4, 1.6)	1'
14	79.4	3.94, dd (8.4, 2.0)		79.4	3.92, dd (8.4, 2.0)	15
15	196.8	9.43, d (2.0)	14	196.8	9.44, d (2.0)	14
1'	168.4			167.7		
2'	128.9			137.4		
3'	138.9	6.10, m		126.8	5.65, dd (3.2, 1.6)	
					6.14, dd (3.2, 1.6)	
14-OCH_3_	56.9	3.10, s	14	57.0	3.09, s	14
2'-Me	20.7	1.88, pentet (1.6)	1', 2'	18.5	1.94, brs	1', 2'
3'-Me	15.9	1.93, dq (7.2, 1.6)	2', 3'			

Note: *^a^* HMBC correlations start from proton(s) to the indicated carbon.

**Table 2:** Cytotoxicity of compounds **1**–**4** against cancer cell lines.

Compounds	IC_50_ (μM)					
	MCF-7	AsPC-1	SW480	HCT116	HepG2	HeLa
**1**	9.5 ± 0.3	14.5 ± 0.9	1.8 ± 0.1	5.9 ± 0.2	20.2 ± 1.1	33.3 ± 2.3
**2**	8.7 ± 0.4	12.1 ± 0.2	0.9 ± 0.1	3.2 ± 0.3	9.9 ± 0.4	23.9 ± 1.2
**3**	9.7 ± 0.7	7.3 ± 0.5	5.2 ± 0.4	9.2 ± 0.6	14.4 ± 1.0	12.3 ± 0.7
**4**	12.7 ± 0.7	4.9 ± 0.2	3.8 ± 0.1	11.4 ± 0.8	27.8 ± 1.4	24.7 ± 0.9
**cisplatin**	13.0 ± 0.6	2.3 ± 0.2	4.8 ± 0.4	3.6 ± 0.1	5.9 ± 0.7	0.89 ± 0.1

Against HCT116 and HepG2 cells, compound **2** showed relatively high cytotoxicity, and against MCF-7 cells all compounds showed moderate cytotoxicity. All of these compounds displayed weak cytotoxicity against HeLa cells, with IC_50_ values (12.3–33.3 μM) compared to that of cisplatin (0.9 μM). Cytotoxicity of sesquiterpenes with α, β-unsaturated lactone structure was well known. Compounds **1** and **2** are uncommon sesquiterpenoids with eight-membered ring and they also showed same type of cytotoxicity as reported [10].

## 3. Experimental Section

### 3.1. General Experimental Procedures

UV spectra were recorded using an Ultraspec 4000 double beam spectrophotometer (Pharmacia Biotech, Cambridge, UK). 1D- and 2D-NMR spectra were obtained on a UNITY INOVA 400 spectrometer (Varian, Palo Alto, CA, USA). Mass spectra were determined on a JMS-AX505WA mass spectrometer (JEOL, Tokyo, Japan). Column chromatography was carried out over silica gel (40–60 μm, Merck, Merck, Kenilworth, NJ, USA), LiChroprep RP-C18 (40–60 μm, Merck) and µ-Bondapak C_18_ column (10 μm, 10 i.d. × 300 mm) (Waters Co., Milford, MA, USA). Fractions obtained from column chromatography were monitored by thin layer chromatography (TLC) (RP-C18 F_254__S_ and silica gel 60 F_254_, Merck). 

### 3.2. Plant Material

The whole plant of *Siegesbeckia glabrescens* Makino (Compositae) was collected from Wan-Do, Jeolla-Namdo Province, Korea in November 2005 and authenticated by Prof. K. S. Yang at the College of Pharmacy, Sookmyung Women’s University (SMU). A voucher specimen (No. SPH 2005007) was deposited in the herbarium of SMU.

### 3.3. Extraction and Isolation

The air-dried material (5 kg) was reflux extracted with methanol (6 × 2 L) to yield after solvent removal a crude methanol extract (578 g), which was successively partitioned twice with EtOAc (3 L) and H_2_O (3 L). The EtOAc soluble fraction (368 g) was subjected to silica gel column chromatography (CC) (13 × 26 cm, 0.063–0.2 mm) eluting with a gradient mixture of CHCl_3_–MeOH (100:1, 70:1, 30:1, 20:1, 12:1, 5:1; 2 L each) to give 26 fractions. Fraction 9 (45 g, V_R_ 5.5–6.0 L) was further fractionated by silica gel column with a gradient elution of CHCl_3_–MeOH (40:1 to 36:1, 2 L each) to afford Fr. 9-4 (3.4 g, V_R_ 1.2–1.6 L). Fr. 9-4 was subjected to an ODS column (5 × 8 cm, 0.040–0.063 mm) eluting with MeOH–H_2_O (1:1) to afford cytotoxic Fr. 9-4-1 (400 mg, V_R_ 1.2–1.4 L) which was subjected to Sephadex LH-20 CC (0.018–0.111 mm) eluted with 70% MeOH to yield pure compound **1** (18 mg, V_R_ 230–290 mL). Fr. 9-4-1-1 (113.6 mg) was subjected to ODS column chromatography eluting with MeOH–H_2_O (1:1.6) to yield compound **2** (11.2 mg, V_R_ 80–120 mL). Fr. 11 (4.0 g, V_R_ 9.1–10.8 L) was separated by a silica gel column chromatography eluting with CHCl_3_–MeOH (40:1) to obtain cytotoxic Fr. 11-9 (234 mg, V_R_ 2.8–3.2 L). Fr. 11-9 (234 mg) was purified by ODS column chromatography eluting with MeOH–H_2_O (1:3) to yield compounds **3** (40 mg, V_R_ 120–145 mL) and **4** (11 mg, V_R_ 210–240 mL). The purities of compounds **1**–**4** were higher than 95% as confirmed by HPLC chromatogram and ^1^H-NMR spectra.

Compound **1**: amorphous solid; ${\left[\alpha \right]}_{D}^{24}$: −96.4, (c 0.005, MeOH), UV (MeOH) λ_max_ (log ε) 244 (3.11), 235 (3.10) nm; IR (CaF_2_, cm^−1^) 3476, 2925, 1764, 1720, 1686. ^1^H- and ^13^C-NMR data, see Table 1; HREIMS *m/z* 390.1685 [M]^+^ (calcd for C_21_H_26_O_7_, 390.1678); EIMS *m/z* 390 [M]^+^.

Compound **2**: amorphous solid; ${\left[\alpha \right]}_{D}^{27}$: −5.7, (c 0.006, MeOH), UV (MeOH) λ_max_ (log ε) 253 (3.20), 245 (3.18) nm; IR (CaF_2_, cm^−1^) 3500, 2931, 1766, 1722, 1686, 1157. ^1^H- and ^13^C-NMR data, see Table 1; HREIMS *m/z* 376.1518 [M]^+^ (calcd for C_20_H_24_O_7_, 376.1522); EIMS *m/z* 376 [M]^+^.

### 3.4. Cytotoxicity Assay

The cytotoxicity of compounds **1**–**4** against human breast cancer (MCF-7), pancreatic cancer (AsPC-1), colon cancers (SW480 and HCT 116), hepatoma (HepG2), and cervical carcinoma (HeLa) were assessed by the MTT method [11]. Cells were plated at a density of 3000 cells/well in a 96 well plate. Cells were incubated with various concentrations of compounds **1**–**4** for 3 days, and then treated with MTT (5 mg/mL) solution for 4 h and lysed with DMSO. Absorbance at 540 nm was measured by using a microplate reader (Molecular Devices, Sunnyvale, CA, USA). Cisplatin (purity > 98%) (Sigma, St. Louis, MO, USA) was used as a positive control.

## Supplementary Materials

EIMS, HREIMS, ^1^H-NMR, ^13^C-NMR, DEPT, COSY, HMBC, and NOESY spectra of compounds **1** and **2** are available as supporting information. Supplementary materials can be accessed at: http://www.mdpi.com/1420-3049/20/02/2850/s1.
