# Supplementary material for: New Cytotoxic Sesquiterpenoids from Siegesbeckia glabrescens
Source: Molecules. 2015 Feb 10;20(2):2850–6. doi: 10.3390/molecules20022850 (PMC6272480; doi:10.3390/molecules20022850)
Supplement: Supplementary file 1 [file molecules-20-02850-s001.pdf]

Supplementary Materials

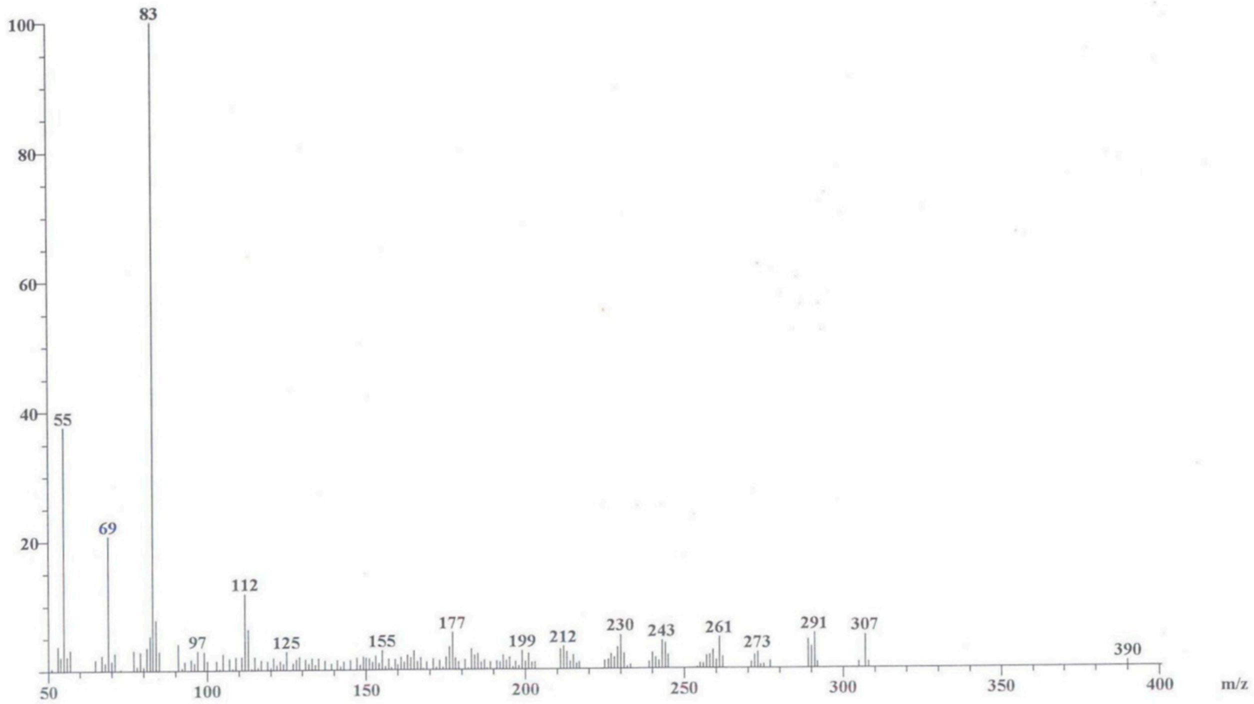

Figure S1. EIMS spectrum of compound **1**.

File: 9441
Sample: sig-Q

Date Run: 12-24-2012
Ionization mode: EI+

Time Run: 15:22:40

Instrument: JEOL JMS600
Inlet: Direct Probe

R.T.: 1:14.05

Scan: 50-65
Base: m/z 343; 59.5%FS

TIC: 4544086

Selected Isotopes : $C_{0\text{-}50}H_{0\text{-}100}O_{0\text{-}10}$

Error Limit : 20 ppm

Measured Mass	*% Base*	*Formula*	*Calculated Mass*	*Error*
**390.1685**	**0.2%**	**$C_{28}H_{22}O_2$**	**390.1620**	**-17.0**
		**$C_{21}H_{26}O_7$**	**390.1678**	**-1.7**

Figure S2. HREIMS spectrum of compound **1**.

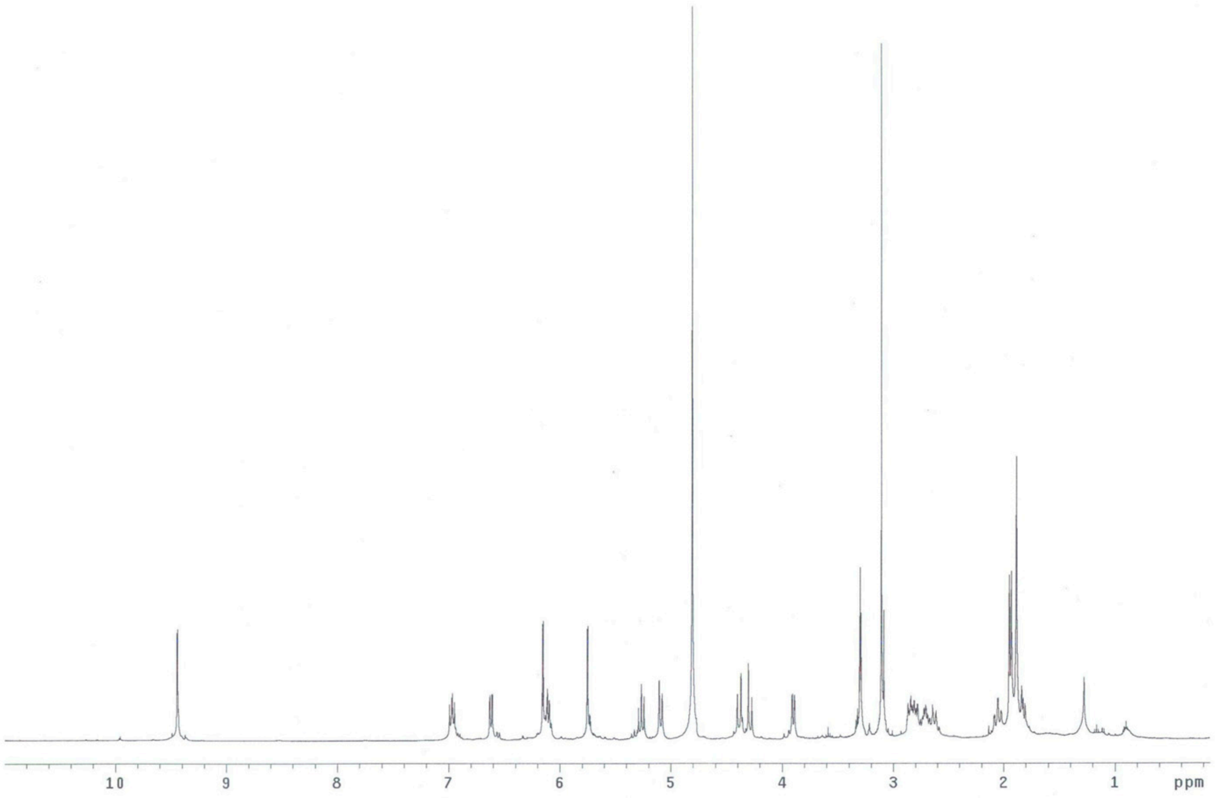

Figure S3. ^{1}H-NMR (400 MHz) spectrum of compound **1** in CD_3OD.

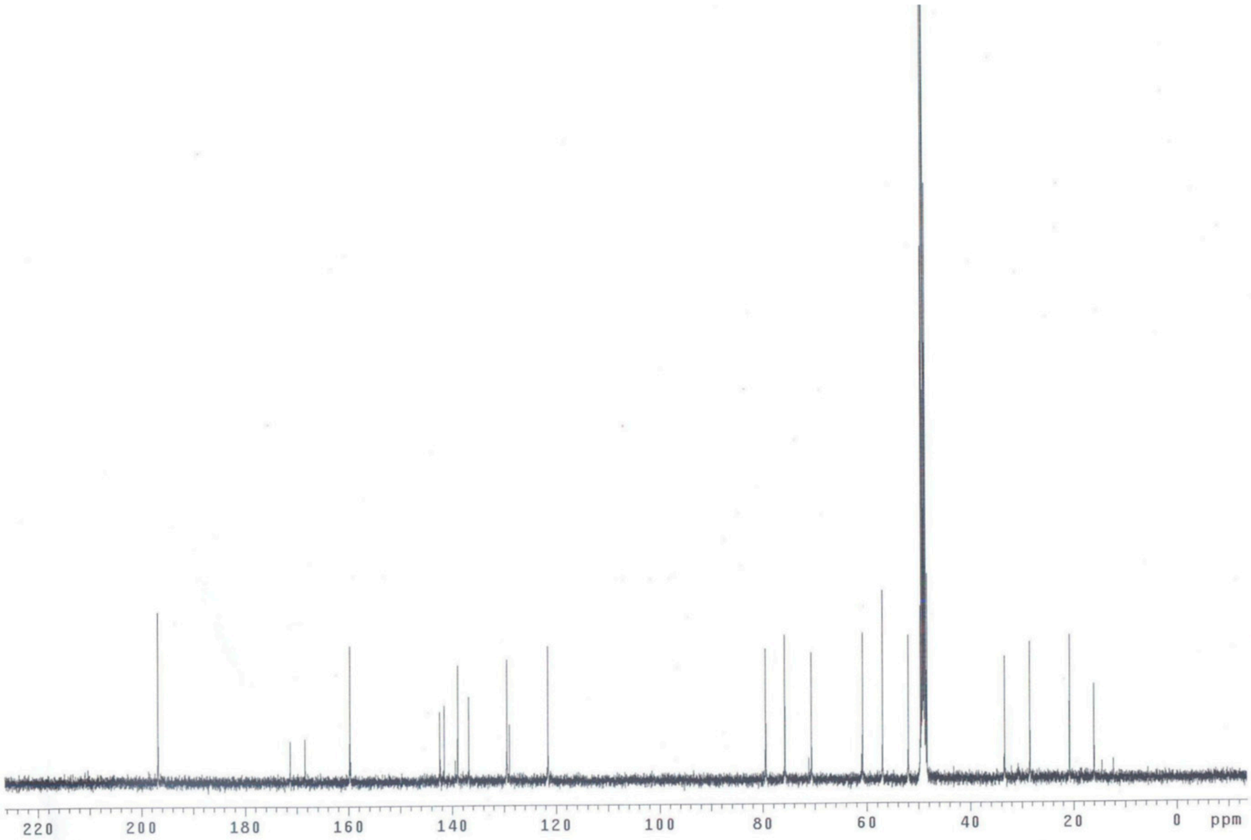

Figure S4. ^{13}C-NMR (100MHz) spectrum of compound **1** in CD_3OD.

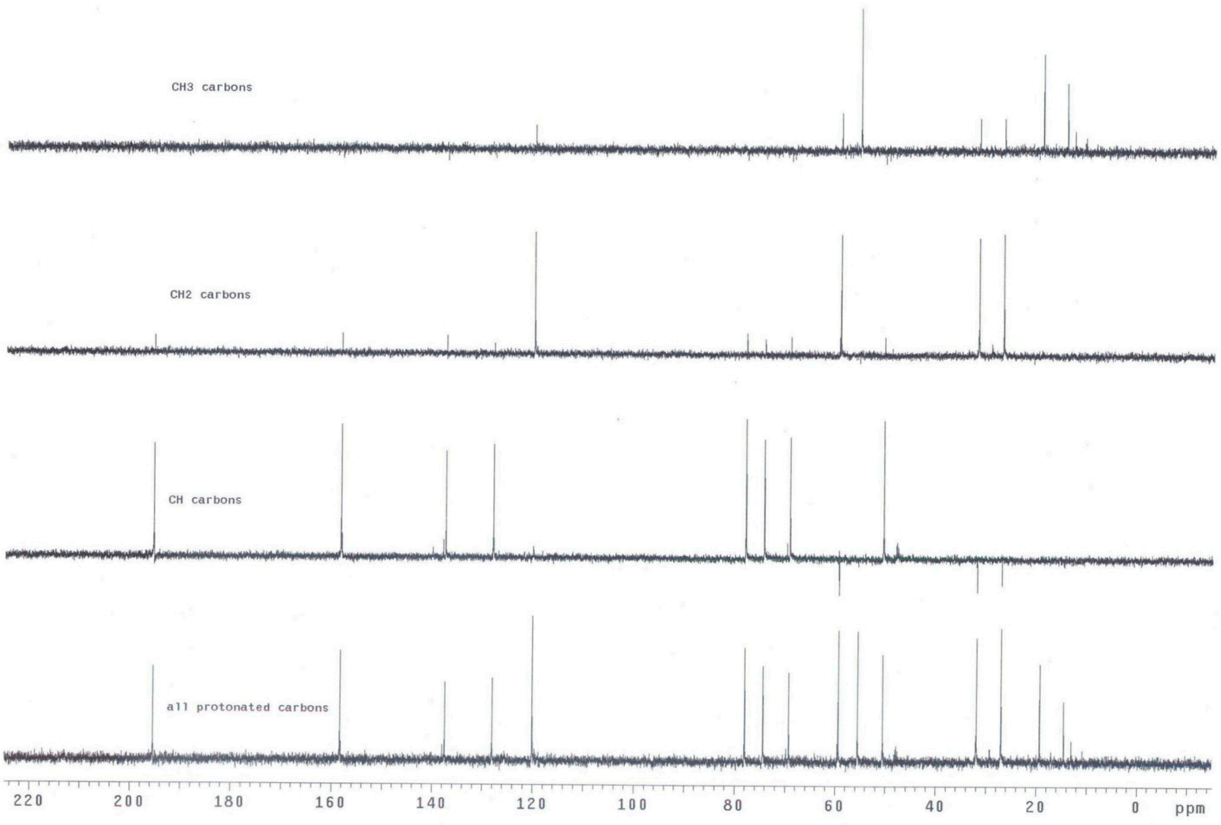

Figure S5. DEPT spectrum of compound **1** in CD_3OD.

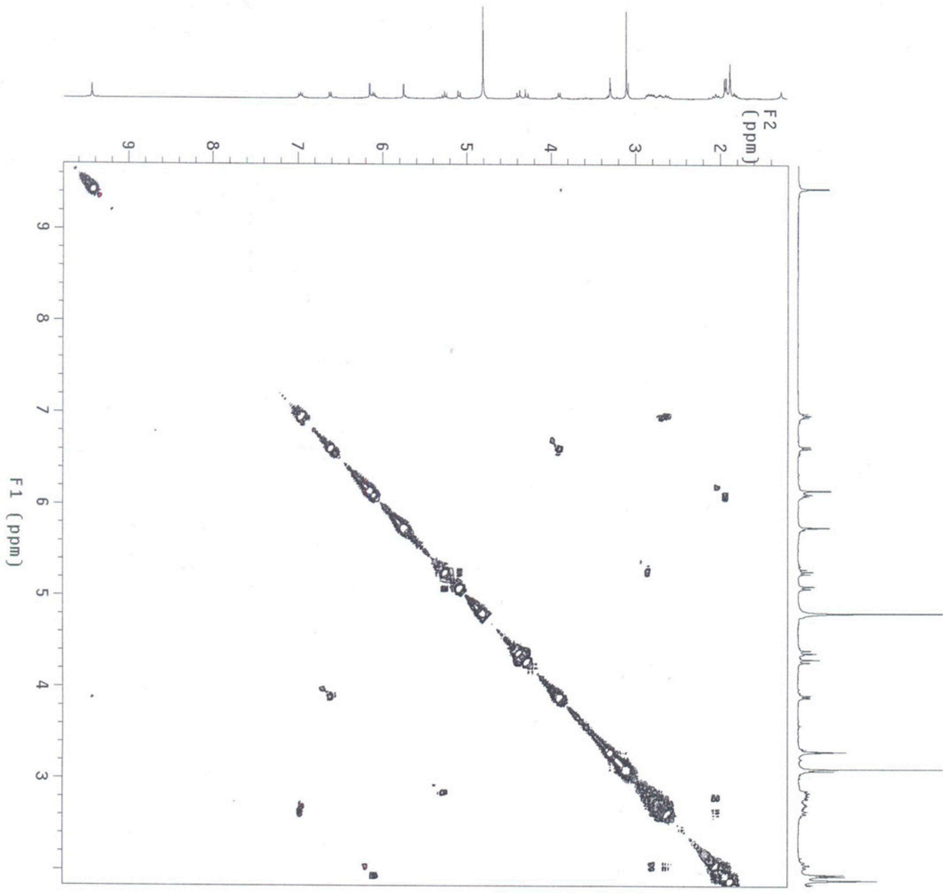

Figure S6. ^{1}H-^{1}H COSY spectrum of compound **1** in CD_3OD.

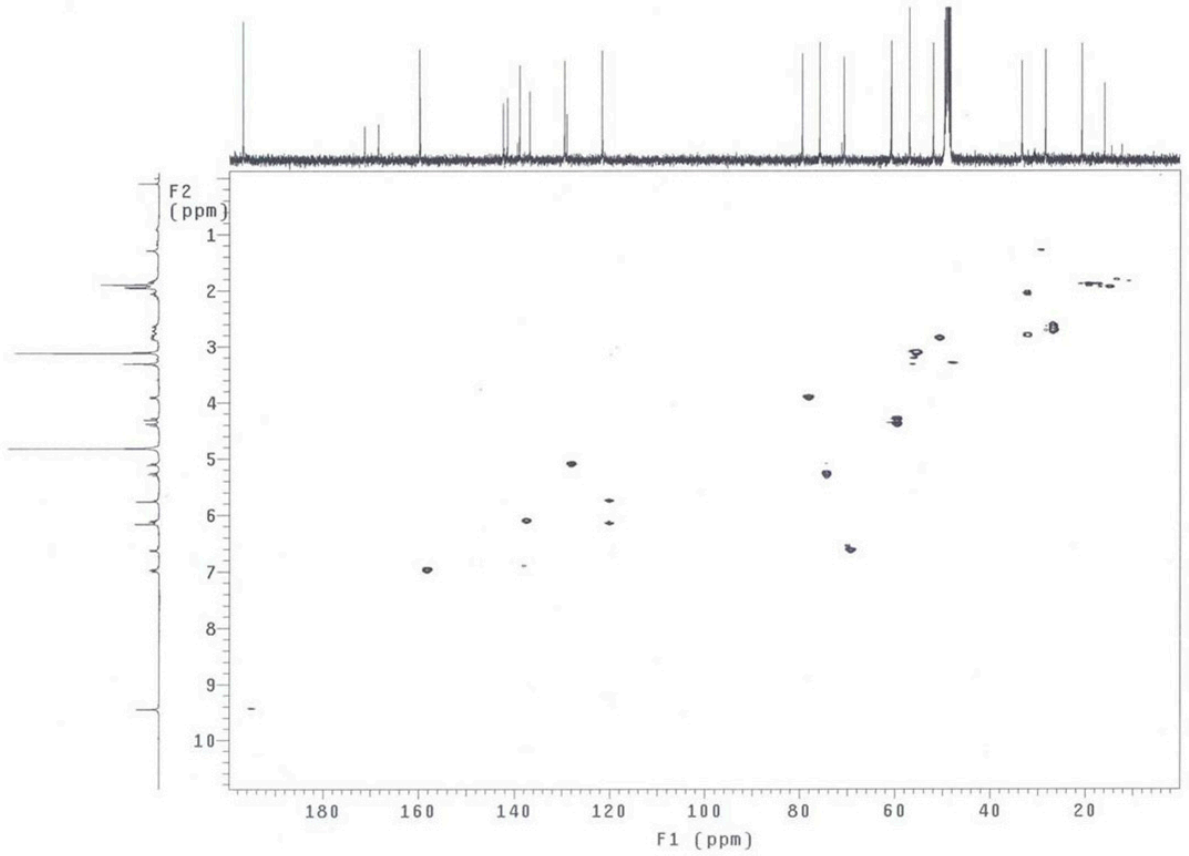

Figure S7. HSQC spectrum of compound **1** in CD_3OD.

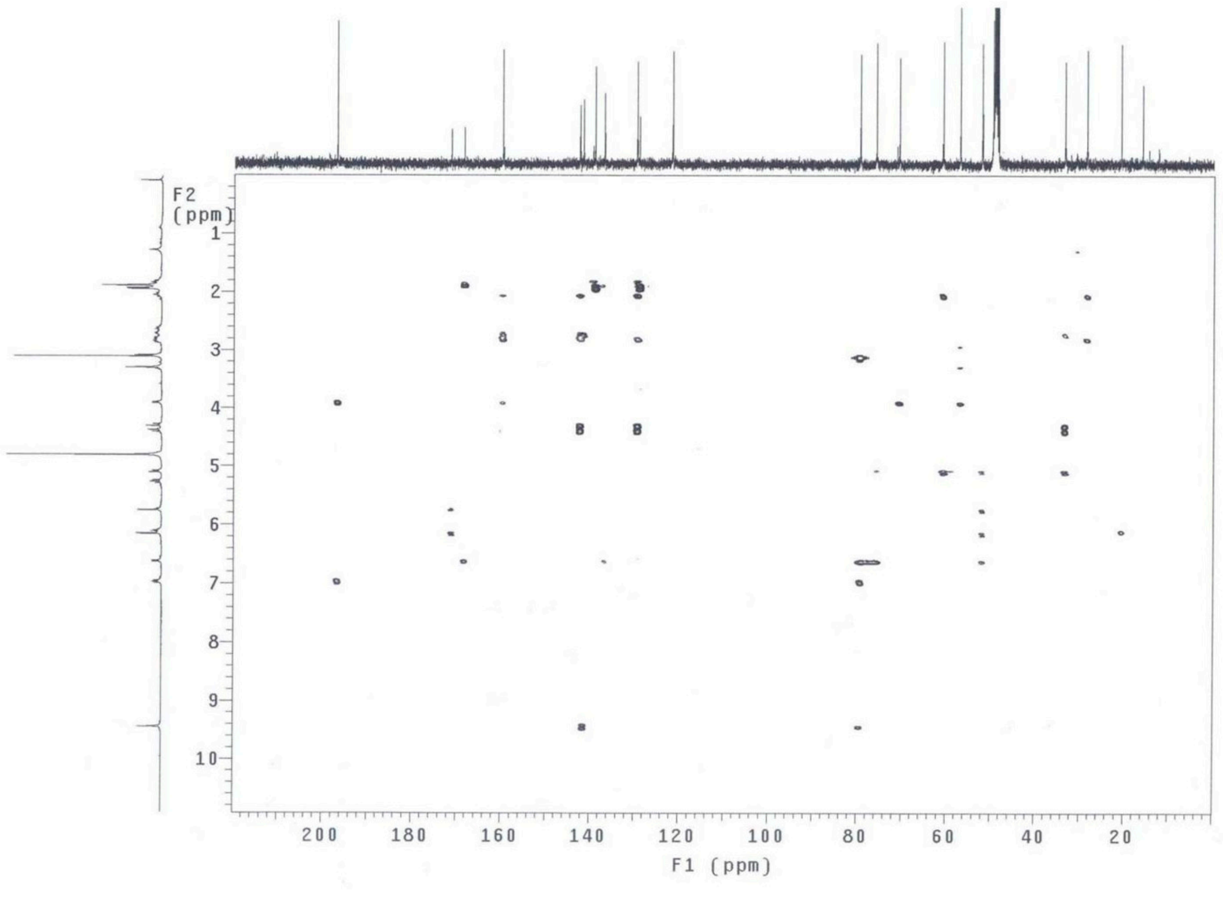

Figure S8. HMBC spectrum of compound **1** in CD_3OD.

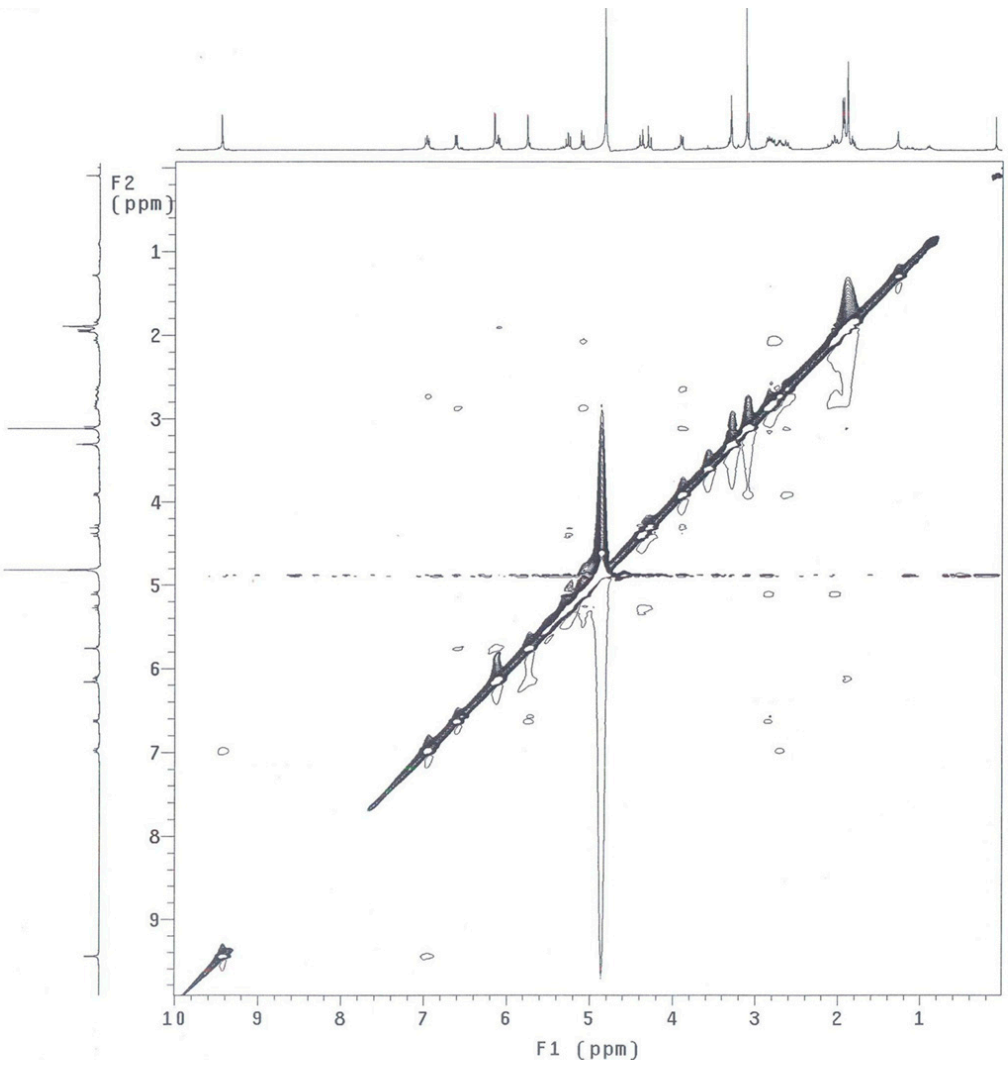

Figure S9. NOESY spectrum of compound **1** in CD_3OD.

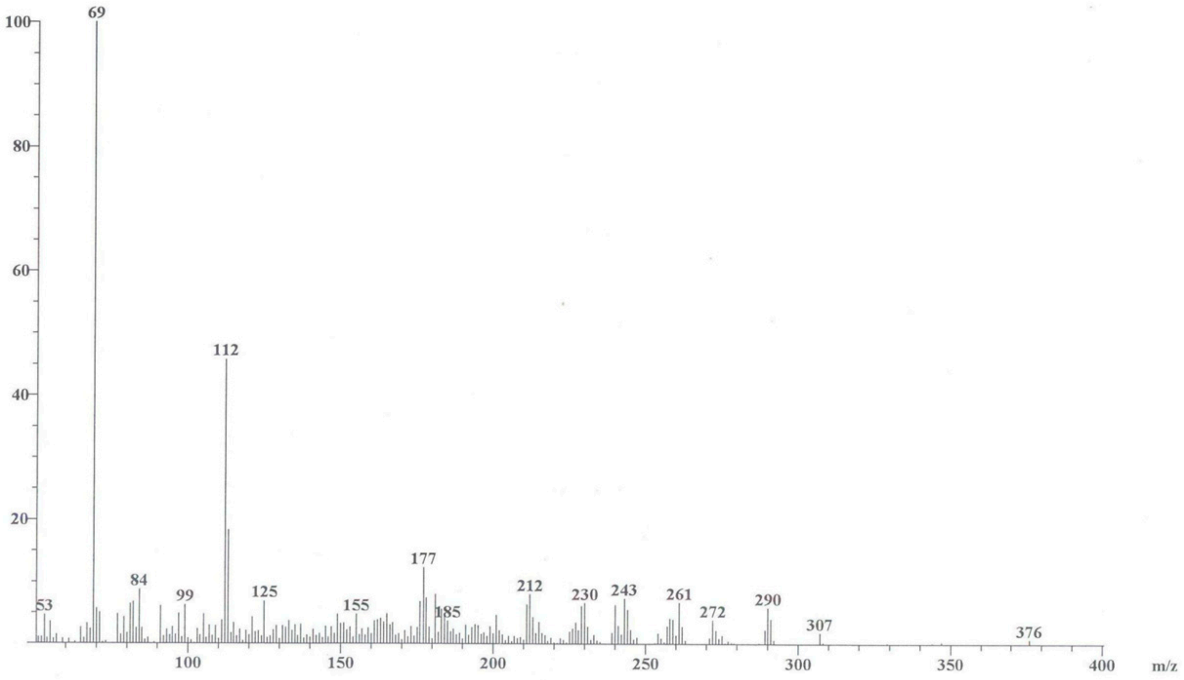

Figure S10. EIMS spectrum of compound **2**.

File: 9442 Date Run: 12-24-2012 Time Run: 15:50:18
Sample: sig-L Ionization mode: EI+

Instrument: JEOL JMS600
Inlet: Direct Probe

R.T.: 2:12.35

Scan: 85-120
Base: m/z 419; 5.6%FS TIC: 1607109

Selected Isotopes : $C_{0\text{-}50}H_{0\text{-}100}O_{0\text{-}10}$ **Error Limit : 20 ppm**

Measured Mass	*% Base*	*Formula*	*Calculated Mass*	*Error*
**376.1518**	**13.6%**	$C_{27}H_{20}O_2$	**376.1463**	**-15.0**
		$C_{20}H_{24}O_7$	**376.1522**	**1.1**

Figure S11. HREIMS spectrum of compound **2**.

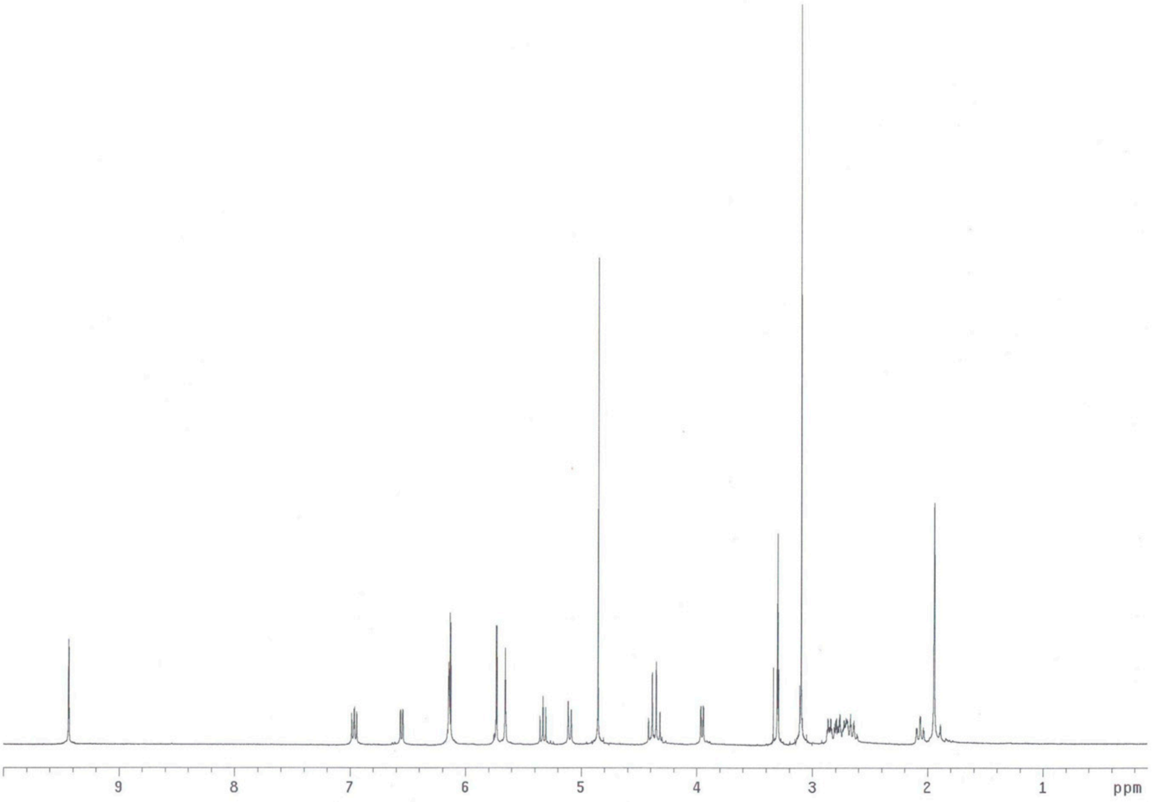

Figure S12. ^{1}H-NMR (400 MHz) spectrum of compound **2** in CD_3OD.

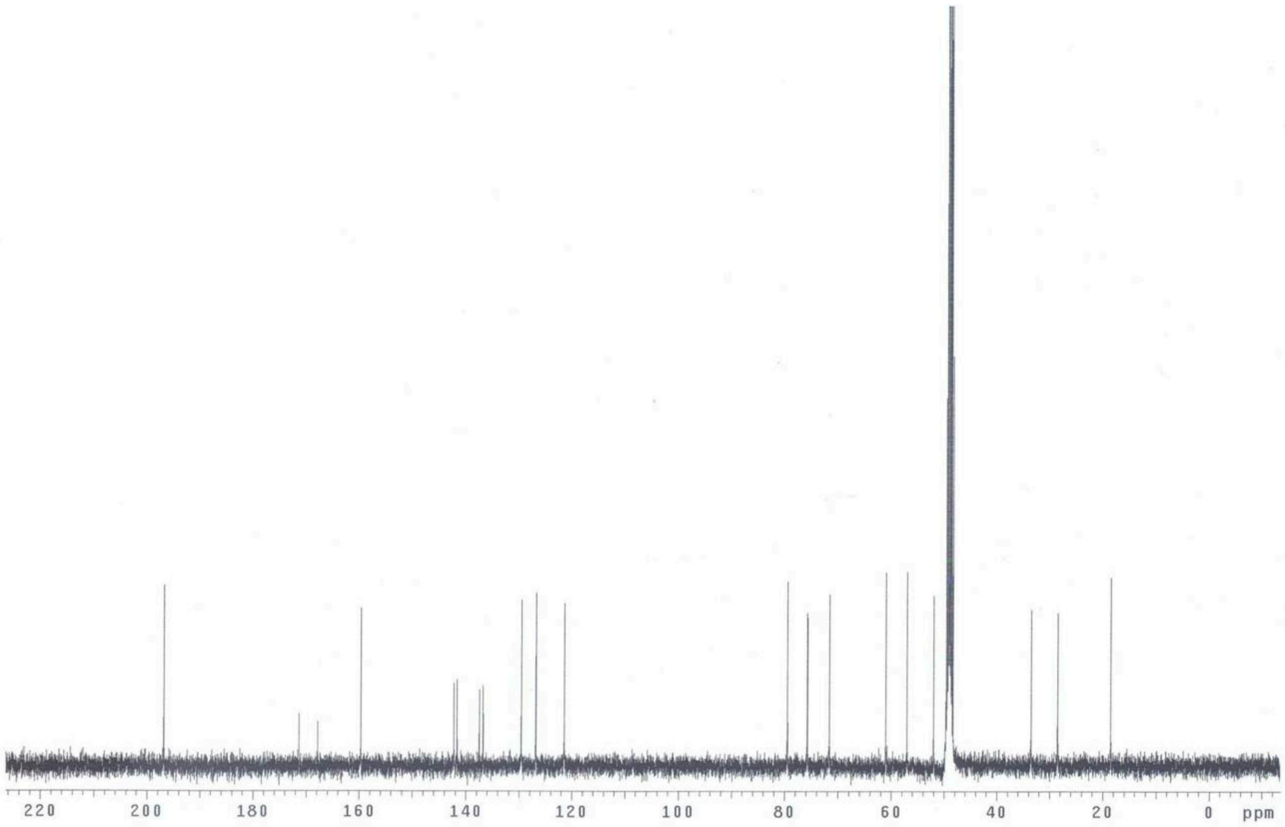

Figure S13. ^{13}C-NMR (100 MHz) spectrum of compound **2** in CD_3OD.

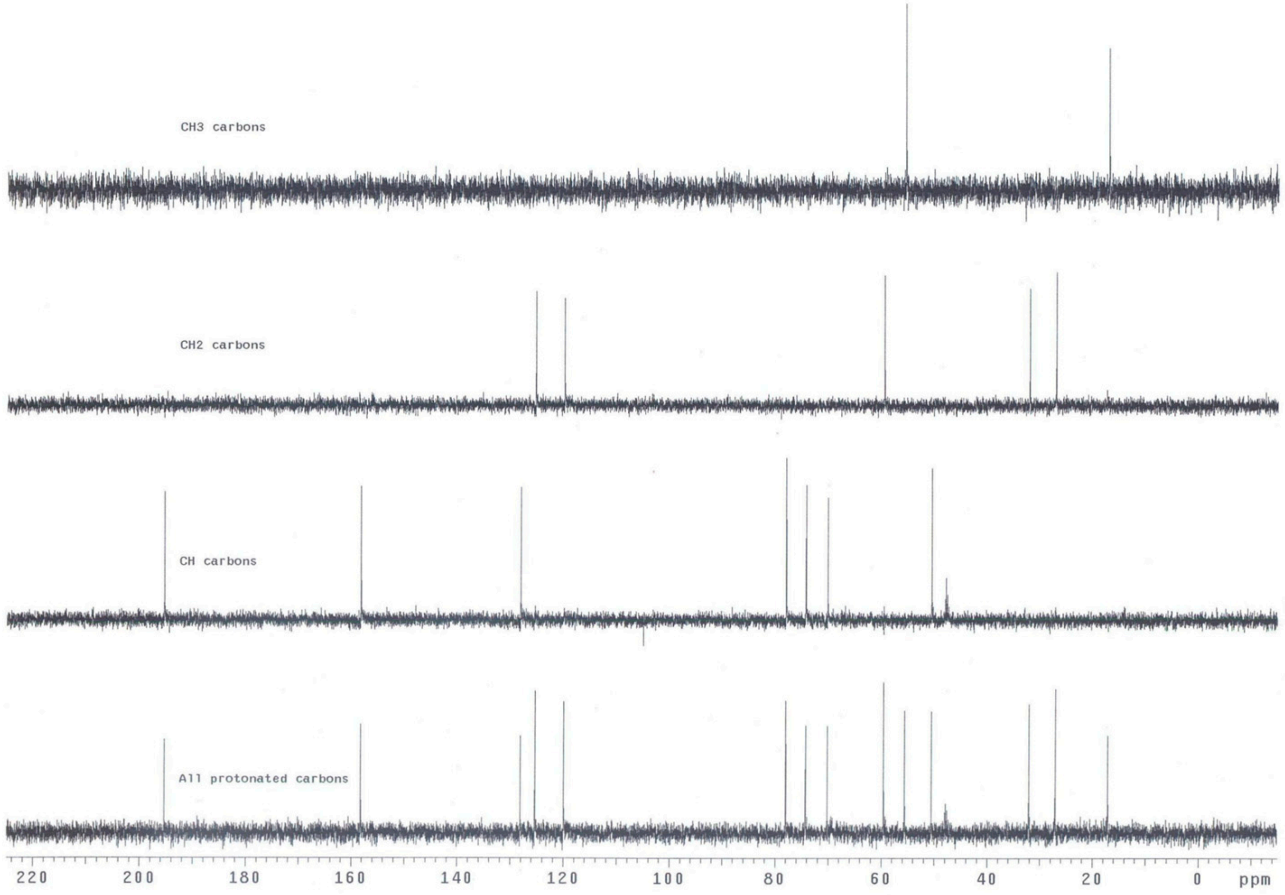

Figure S14. DEPT spectrum of compound **2** in CD_3OD.

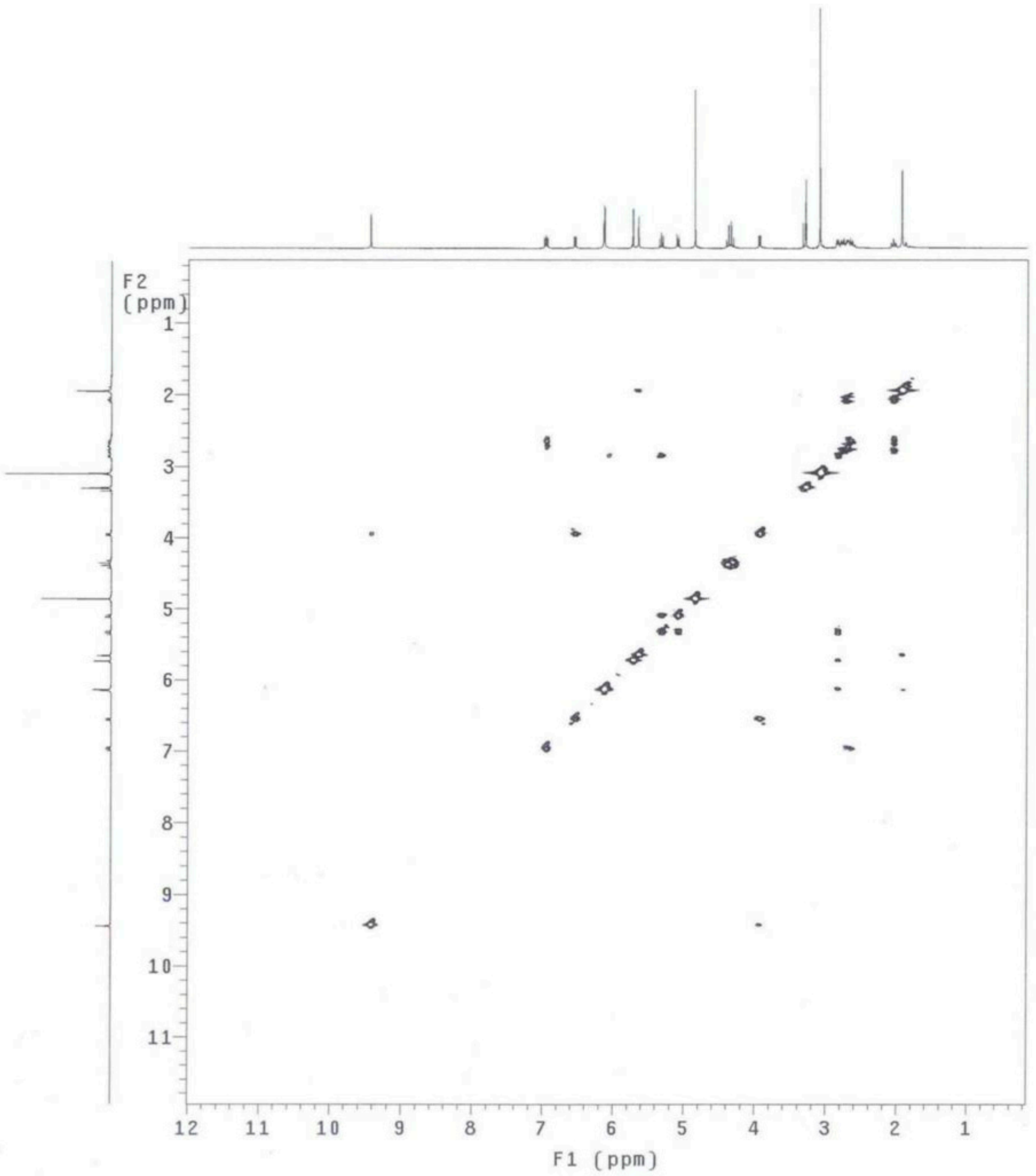

Figure S15. ^{1}H-^{1}H COSY spectrum of compound **2** in CD_3OD.

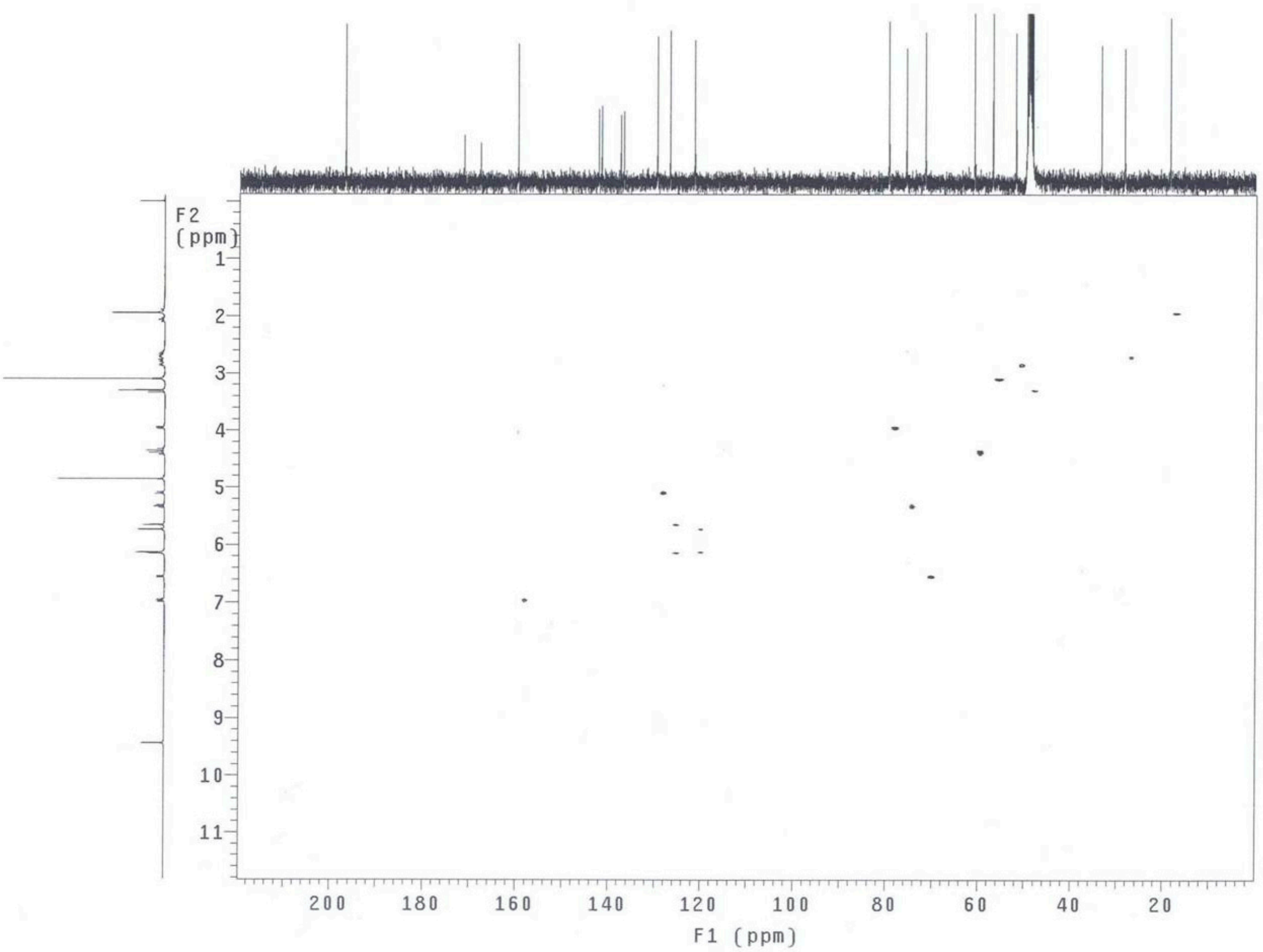

Figure S16. HSQC spectrum of compound **2** in CD_3OD.

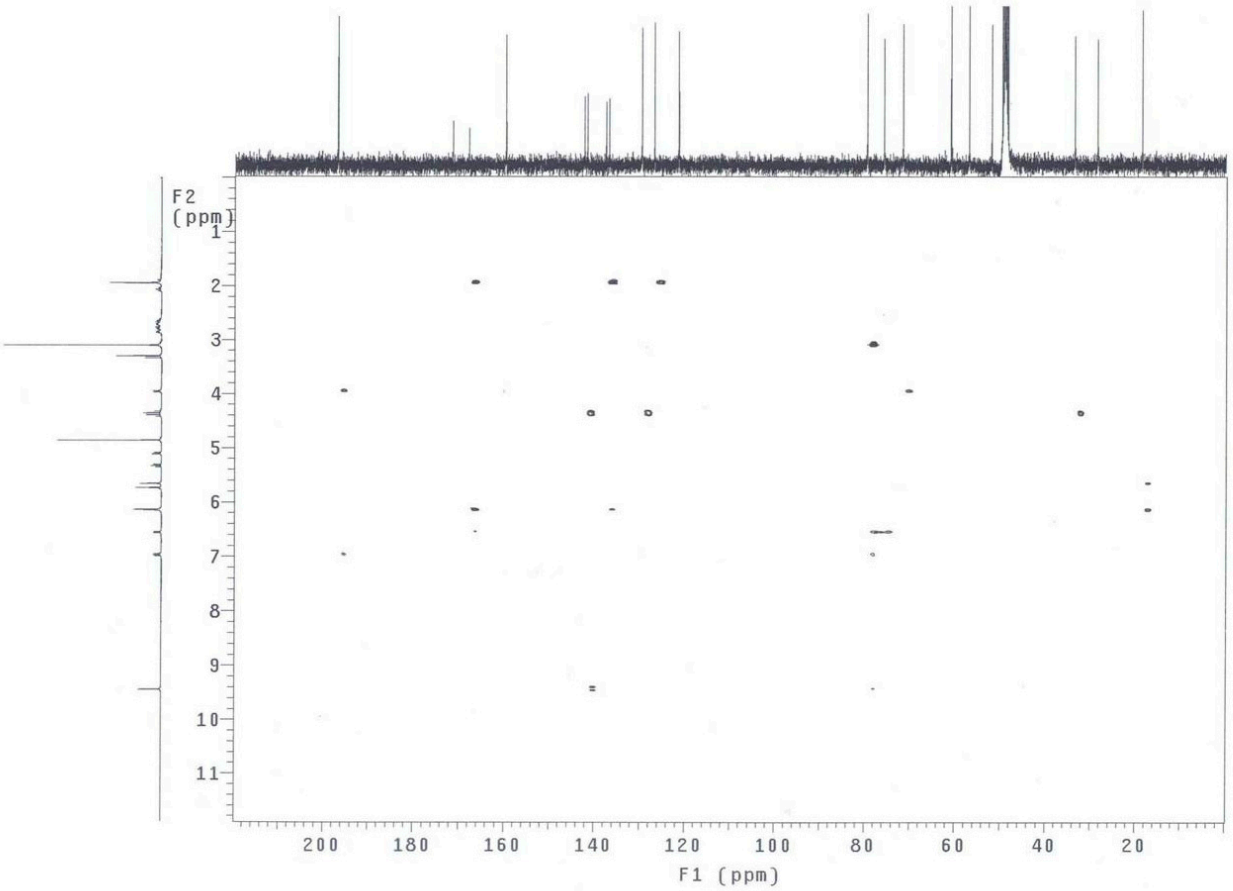

Figure S17. HMBC spectrum of compound **2** in CD_3OD.

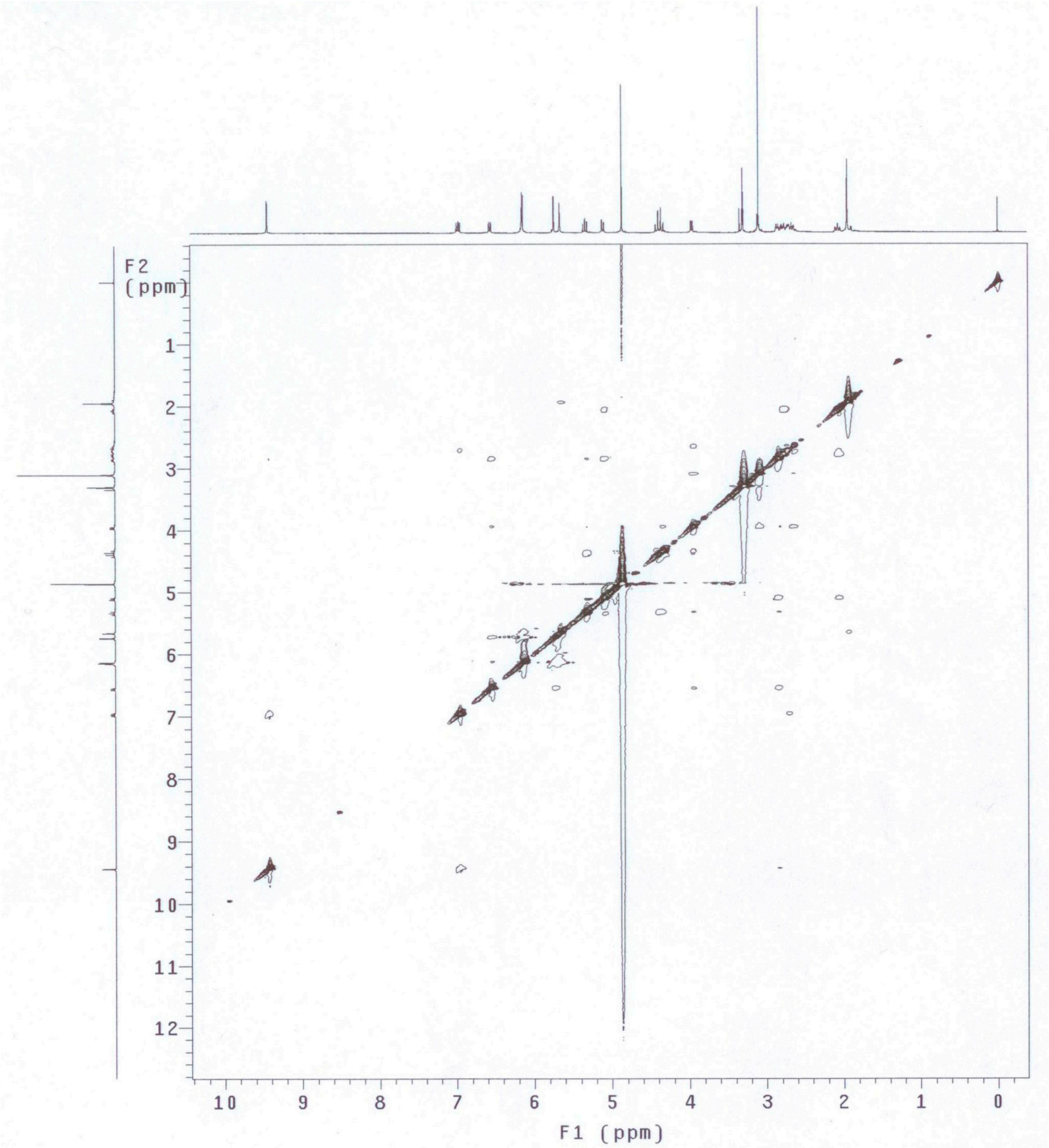

Figure S18. NOESY spectrum of compound **2** in CD_3OD.
